# Variability of Metabolite Levels Is Linked to Differential Metabolic Pathways in Arabidopsis's Responses to Abiotic Stresses

**DOI:** 10.1371/journal.pcbi.1003656

**Published:** 2014-06-19

**Authors:** Nadine Töpfer, Federico Scossa, Alisdair Fernie, Zoran Nikoloski

**Affiliations:** 1 Systems Biology and Mathematical Modeling Group, Max Planck Institute of Molecular Plant Physiology, Potsdam, Germany; 2 Central Metabolism Group, Max Planck Institute of Molecular Plant Physiology, Potsdam, Germany; 3 Consiglio per la Ricerca e la Sperimentazione in Agricoltura, Centro di ricerca per l'Orticoltura, Pontecagnano (Salerno), Italy; North Carolina State University, United States of America

## Abstract

Constraint-based approaches have been used for integrating data in large-scale metabolic networks to obtain insights into metabolism of various organisms. Due to the underlying steady-state assumption, these approaches are usually not suited for making predictions about metabolite levels. Here, we ask whether we can make inferences about the variability of metabolite levels from a constraint-based analysis based on the integration of transcriptomics data. To this end, we analyze time-resolved transcriptomics and metabolomics data from *Arabidopsis thaliana* under a set of eight different light and temperature conditions. In a previous study, the gene expression data have already been integrated in a genome-scale metabolic network to predict pathways, termed modulators and sustainers, which are differentially regulated with respect to a biochemically meaningful data-driven null model. Here, we present a follow-up analysis which bridges the gap between flux- and metabolite-centric methods. One of our main findings demonstrates that under certain environmental conditions, the levels of metabolites acting as substrates in modulators or sustainers show significantly lower temporal variations with respect to the remaining measured metabolites. This observation is discussed within the context of a systems-view of plasticity and robustness of metabolite contents and pathway fluxes. Our study paves the way for investigating the existence of similar principles in other species for which both genome-scale networks and high-throughput metabolomics data of high quality are becoming increasingly available.

## Author Summary

Organisms are usually exposed to changing environments and balance these perturbations by altering their metabolic state. Gaining a deeper understanding of metabolic adjustment to varying external conditions is important for the development of advanced engineering strategies for microorganisms as well as for higher plants. One tool which is particularly suited for investigating these processes is genome-scale metabolic models. These large-scale representations of the underlying metabolic networks enable the integration of experimental data and application of constrain-based mathematical approaches to estimate flux rates through the chemical reactions of the network under different environmental scenarios. However, for most of these approaches the assumption of a steady-state (flux balance) is indispensable and therefore precludes the prediction of metabolite concentrations. Here, we present a data-driven observation that relates results from a flux-centric constraint-based approach that is based on transcriptomics data to metabolite levels from the same experiments. Our observations suggest that constraint-based modeling approaches in combination with high-throughput data can be used to infer regulatory principles about the plasticity and robustness of metabolic behavior from the stoichiometry of the underlying reactions alone.

## Introduction

Organisms, especially plants, are exposed to almost perpetually changing environments (*e.g.*, light intensity and quality, nutrient and water supply) to which they respond by readjusting their cellular setup to efficiently utilize available resources and to ensure viability [1]–[6]. These transitions are often systemic in that they affect almost all levels of cellular organization, starting from gene expression to protein abundances and metabolite levels [7]–[9]. Therefore, a systems-based analysis is particularly suited for understanding the responses of plants to changes in the environment. Such an approach offers the possibility to integrate data which were simultaneously collected across different cellular levels to identify dependence between processes and to aid in testing hypotheses concerning the behavior of individual components or pathways.

Constraint-based approaches provide a modeling framework which is particularly amenable for systems-based analyses, since they not only allow for the integration of high-throughput data, but also rely almost solely on the stoichiometry of the reactions included in the models. For instance, with the help of Flux Balance Analysis (FBA, for details see Material and Method section) [10], [11] condition-specific steady-state flux distributions and growth capabilities can be readily predicted [12]. Moreover, recent studies have established that integration of high-throughput data can narrow down the space of feasible flux distributions and, therefore, results in improved predictions of biomass or contributes to more physiologically realistic engineering strategies [13]–[15]. The existing constraint-based approaches, which integrate data rely mostly on transcriptomics data and assume a relationship between the expression level of a given gene and the flux boundaries of the corresponding reaction in the metabolic network [14], [16], [17].

However, one of the main drawbacks of most constraint-based approaches lies in the nature of their problem formulation, *i.e.*, the steady-state assumption, which precludes the integration and prediction of metabolite levels (detailed in the Materials and Methods section). Therefore, these approaches usually neglect the metabolome *i.e.*, the levels of all considered metabolites which, along with reaction fluxes, act as one of the most informative indicators of the cellular metabolic state [18]. Existing attempts to integrate metabolite levels/concentrations into constraint-based approaches are restricted to predictions of reaction directionality via thermodynamic analysis [19]–[21] or require relaxation of the steady-state assumption [22]. In the current study, we ask whether (and if so, to what extent) we can make inferences about metabolite levels from a constraint-based analysis that is based on the integration of transcriptomics data.

In order to do so, we extend a previous study in which we used microarray data from *Arabidopsis thaliana* [23],[24] and integrated them in a metabolic network [25] to predict flux capacities for a large set of pathways under eight different light- and temperature conditions [26]. Furthermore, to make statistical statements, we compared the flux capacity profiles to those obtained from a biochemically meaningful data-driven null model. Based on this, we defined a pathway to be differential under a given condition if it exhibits a flux capacity profile that has an average absolute 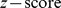 greater that 2 with respect to the null model. Moreover, we introduced the concept of metabolic sustainers and modulators. Sustainers are metabolic functions that are differentially up-regulated with respect to the null model and sustain a certain functioning, whereas modulators are differentially down-regulated [23], [24] to control a certain flux and modulate affected processes. A more detailed description of this study is given in the Results section.

Here, we present observations that link predictions made from the integration of transcriptomics data to metabolomics data from the same experiment. By doing so, we bridge the gap between flux- and metabolite-centric approaches. Most importantly, our findings demonstrate that under certain conditions, metabolites acting as substrates in pathways defined as modulators or sustainers of the metabolic state show a significantly lower temporal variation in comparison to the remaining metabolites. These observations are discussed within the context of a systems-view of plasticity and robustness of metabolite content as well as reaction/pathway fluxes. Taken together, our results demonstrate the power of transcriptomic data in predicting metabolic behavior in large-scale models and suggest an underlying regulatory principle governing metabolic stability.

## Results

### Data integration in a constraint-based model to predict condition-specific differential metabolic functions

FBA's objective function has a large effect on the predicted flux distribution [27]. For microorganisms under ambient conditions, the maximization of growth is a widely used cellular objective [28], [29]. However, when modeling plants metabolism this assumed objective does not necessarily hold true. Plants are more complex than microorganisms. They have multiple compartments within the cell, different cell types, several tissues and organs, which make it difficult to define a single objective function for the entire plant. Defining such an objective becomes even more challenging under stress conditions which have been shown to drastically alter plant's cellular chemical composition (see [5], [30], [31] and references therein).

In consideration of the absence of a reasonable biological objective function for plants experiencing stress, in our previously presented approach [23] we did not attempt to make predictions about actual fluxes through a metabolic pathway but rather aimed at predicting flux capacities. These capacities are derived from the integration of transcriptomics data into a large-scale metabolic model and represent maximum fluxes which certain pathways can carry under certain environmental conditions. While this concept can also be applied to single reactions of a network, we relied on the investigation of the functional units, the pathways, or in a more generic terminology, the metabolic functions.

We employed a transcriptomics dataset which captures the temporal response of *Arabidopsis thaliana* to eight different light and temperature conditions [26] and used the data to constrain the upper and lower flux boundaries of the reactions based on the E-Flux method [16] in a recent compartmentalized genome-scale model of *Arabidopsis* [25]. Subsequently, we predicted the flux capacities through a set of 167 metabolic functions, from primary and secondary metabolism, for each time-point and each condition.

Furthermore, to make statistical statements about the metabolic functions under consideration we compared the resulting flux capacities to predictions from a null model as a reference state. This analysis was motivated by the need to determine behavior of a metabolic function in a particular condition irrespective of an artificially placed reference state, which may not be representative for the “naturally occurring environment” which the plant experiences in the field. The employed null model was based on the permutation of the assigned flux boundaries while keeping thermodynamic and exchange constraints unaltered. In this manner, we circumvented issues with the selection of a reference state and relied on the average behavior determined solely by the network structure and the imposed flux boundaries. We re-computed the flux capacities from the null model for 100 repetitions for each time point and condition. Based on this, a metabolic function was deemed differential if it showed an absolute 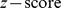 greater than 2 with respect to the expectation from the null model in at least one but not all conditions under consideration. Pathways that were differentially up-regulated were termed sustainers—sustaining a certain metabolic functioning, while those that were differentially down-regulated were referred to as modulators—modulating a certain metabolic functioning. A complete list of these pathways and their classification under the eight conditions considered is given in [23].

### Metabolites in metabolic functions

The working hypothesis of this study was motivated by the following: The determined modulators and sustainers exhibit flux capacities significantly different from the capacities expected by the null model. Furthermore, we observed that the direction of the differential behavior is unaltered across environmental conditions (*i.e.*, differential metabolic functions are robust, or in genetic terminology, canalized [32], [33]). Therefore, we expected the likelihood for this observation to increase if the metabolites participating in these differentially behaving functions show persistently smaller fluctuations compared to metabolites involved in other functions. To test this hypothesis, we analyzed the metabolite profiles that were collected alongside with the transcriptomics data in the same experiments [26]. A schematic representation of the overall workflow from the data mapping and integration to the statistical analysis is provided in Figure 1. For each of the eight environmental conditions and set of investigated functions, we categorized the 65 mapped metabolites (see Materials and Methods) according to the following criteria: (1) participation in (non-)differentially behaving metabolic function and (2) metabolite, substrate, or product of a pathway. In addition, we made the distinction between substrates/products and initial substrates/initial products of the pathway.

**Figure 1:**
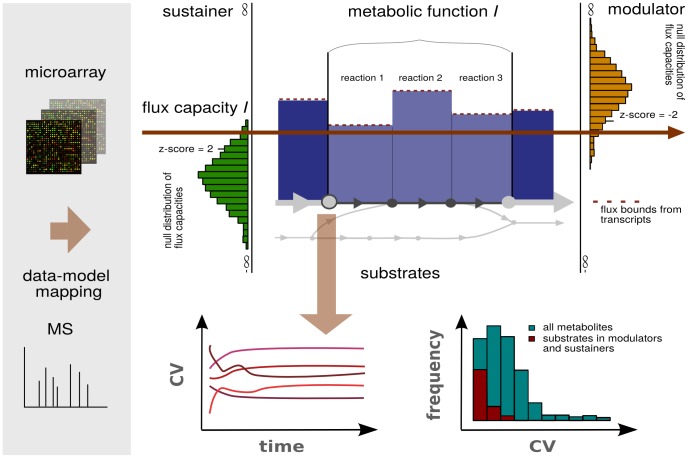
Schematic representation of the analysis framework. Transcriptomics and metabolomics data capturing *Arabidopsis thaliana*'s temporal response to eight different environmental conditions (combinations of different light and/or temperature regimes) are collected for a time-series of 24 hours. The transcriptomic data are used to constrain flux boundaries of the respective reactions in a large-scale network by assuming a correlation between the transcript abundance and the upper flux boundary through the respective enzyme-catalyzed reaction. Based on a model with randomized flux boundaries (null model), pathways are classified as differential for a given condition if they exhibit an absolute 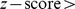
2. Differentially up-regulated (down-regulated) pathways are termed sustainers (modulators) of the metabolic state, respectively. Independently from this categorization, the temporal variation of the metabolite profiles was determined. Under certain conditions, substrates in the differential pathways exhibit a significantly lower temporal variation with respect to other groups of metabolites.

**Figure 2:**
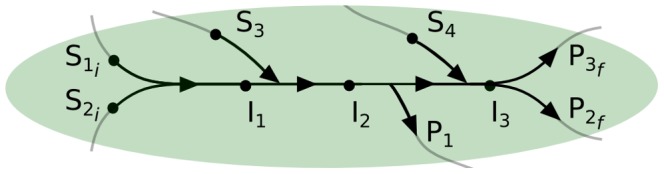
Representation of a metabolic function and its metabolites. Shown is a metabolic pathway with four reactions, represented by the arrows. The dots represent the metabolites, which are categorized as substrates 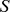, intermediates 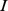 and products 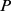 with the subgroup of initial substrates 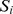 and final products 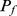.

We defined a substrate of a pathway as any metabolite that acts as a substrate in a reaction involved in the pathway but not as product/intermediate of the same pathway. Furthermore, an initial substrate is a substrate in the first reaction of the pathway. In an analogous manner: a product of a given pathway is defined as any metabolite acting as a product in a reaction of the pathway but not as a substrate/intermediate of the pathway. A product of the last reaction of the pathway is defined as a final product. For the metabolic function in Figure 2, 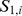, 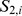, 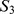 and 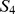 are substrates, while 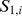 and 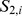 are initial substrates; moreover, 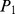,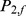 and 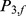 are products, while 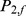 and 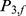 are final products.

### Variability of substrates in differential metabolic functions

We calculated the variability of each considered metabolite over the time course following the perturbation by calculating the coefficient of variation (CV) as described in the Materials and Methods section. In order to determine differences in the distribution of CVs over the considered categories of metabolites we employed the *Wilcoxon* rank-sum test (which also is applicable to non-normal distributions) at a significance level of 0.05. We considered the distribution of CVs across all metabolites, across products only, and across substrates only, in the following six comparisons of groups: modulators vs. all metabolites, sustainers vs. all metabolites, differentially behaving functions (*i.e.*, modulators and sustainers) vs. all metabolites, modulators vs. non-modulators, sustainers vs. non-sustainers, and differentially behaving functions vs. non-differentially behaving functions. As shown in Table 1, this summed up to a total of 144 statistical tests for three categorizations of metabolites over six groupings under eight conditions. A list containing the numbers of tested metabolites for each scenario is given in the Supporting Information S1.

**Table 1:** Significance test for the coefficients of variation (CVs) for different groups of *metabolites*, or *products*, or *substrates* across the considered environmental conditions.

Metabolites/condition	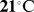 - 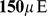	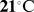 - dark	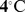 - 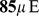	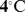 - dark	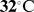 - 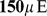	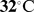 - dark	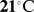 - 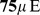	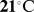 - 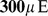
modulator vs. all metabolites	0.238	0.369	0.487	0.158	0.056	0.533	0.145	**0.040**
sustainer vs. all metabolites	0.404	0.338	0.428	0.136	0.279	0.578	0.175	**0.024**
differential vs. all metabolites	0.303	0.264	0.330	0.086	0.067	0.512	0.090	**0.019**
modulator vs. non-modulator	0.427	0.370	0.504	0.105	0.101	0.408	0.420	0.162
sustainer vs. non-sustainer	0.606	0.369	0.489	0.098	0.425	0.377	0.383	0.086
differential vs. non-differential	0.590	0.272	0.364	0.051	0.155	0.311	0.401	0.108
**Products/condition**								
modulator vs. all metabolites	0.283	0.665	0.760	0.282	0.298	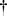	0.416	0.151
sustainer vs. all metabolites	0.397	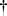	0.213	0.233	0.268	0.563	0.369	0.199
differential vs. all metabolites	0.366	0.715	0.509	0.197	0.215	0.563	0.362	0.178
modulator vs. non-modulator	0.469	0.605	0.761	0.136	0.479	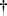	0.862	0.328
sustainer vs. non-sustainer	0.608	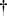	0.244	0.182	0.376	0.519	0.729	0.311
differential vs. non-differential	0.687	0.668	0.526	0.084	0.448	0.519	0.901	0.435
**Substrates**/**condition**								
modulator vs. all metabolites	0.104	0.129	0.248	0.164	**0.030**	0.533	0.072	**0.019**
sustainer vs. all metabolites	0.366	0.338	0.411	0.329	0.282	0.128	0.101	**0.033**
differential vs. all metabolites	0.146	0.075	0.125	0.144	**0.033**	0.228	**0.026**	**0.008**
modulator vs. non-modulator	0.081	0.083	0.224	**0.046**	**0.037**	0.294	0.088	0.104
sustainer vs. non-sustainer	0.371	0.273	0.405	0.195	0.291	**0.046**	0.073	0.115
differential vs. non-differential	0.124	**0.043**	0.108	**0.033**	**0.046**	0.059	**0.029**	0.067

Given are *Wilcoxon*-test 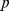-values from testing for significantly lower CVs for ***metabolites*** or ***products*** or ***substrates*** in the given six comparisons, respectively. A 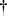 denotes that the sample size was too small for a statistic test (0 or 1 product in the respective group). The table entries in bold mark the significant comparisons at level 0.05.

First, considering the group of all metabolites (Table 1 top), we found the mean CV of metabolites involved in differential metabolic functions to be smaller in comparison to the mean CV of all metabolites under one conditions, *i.e.*, high-light (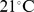 and 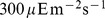, 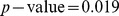). This was also the case when considering the mean CV of metabolites in modulators and sustainers, separately (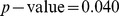 and 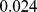, respectively). Secondly, analyzing the group of products of the pathways, we did not observe any significant differences in the CVs in any of the tested groups under any of the eight conditions (Table 1 middle). In contrast, investigating the third group—the substrates (Table 1 bottom) — we found the mean CV of the substrates in modulators to be significantly smaller than the mean CV of all metabolites under two conditions, *i.e.*, 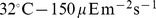 (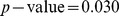) and 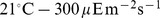 (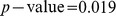). Moreover, the mean CV of the substrates in sustainers was significantly smaller than the mean CV of all metabolites under one condition, *i.e.*, 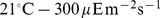 (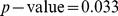). Altogether, the mean CV of the substrates in differentially behaving metabolic functions was significantly smaller than the mean CV of all metabolites under three conditions, namely, under 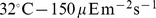 (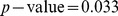), 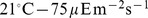 (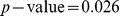), and 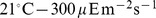 (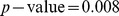). Furthermore, the mean CV of the substrates in modulators was significantly smaller than the mean CV of substrates in non-modulators under two conditions, *i.e.*, 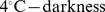 (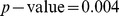) and 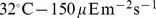 (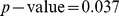), while the mean CV of sustainers was significantly smaller than the mean CV of substrates in non-sustainer under 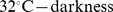 (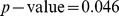). Finally, the mean CV of the substrates in differentially behaving metabolic functions was significantly smaller than the mean CV of substrates in all non-differential functions under four conditions, namely under 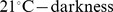 (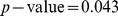), 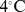-darkness (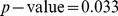), 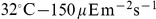 (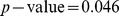), and 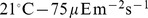 (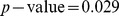).

**Figure 3:**
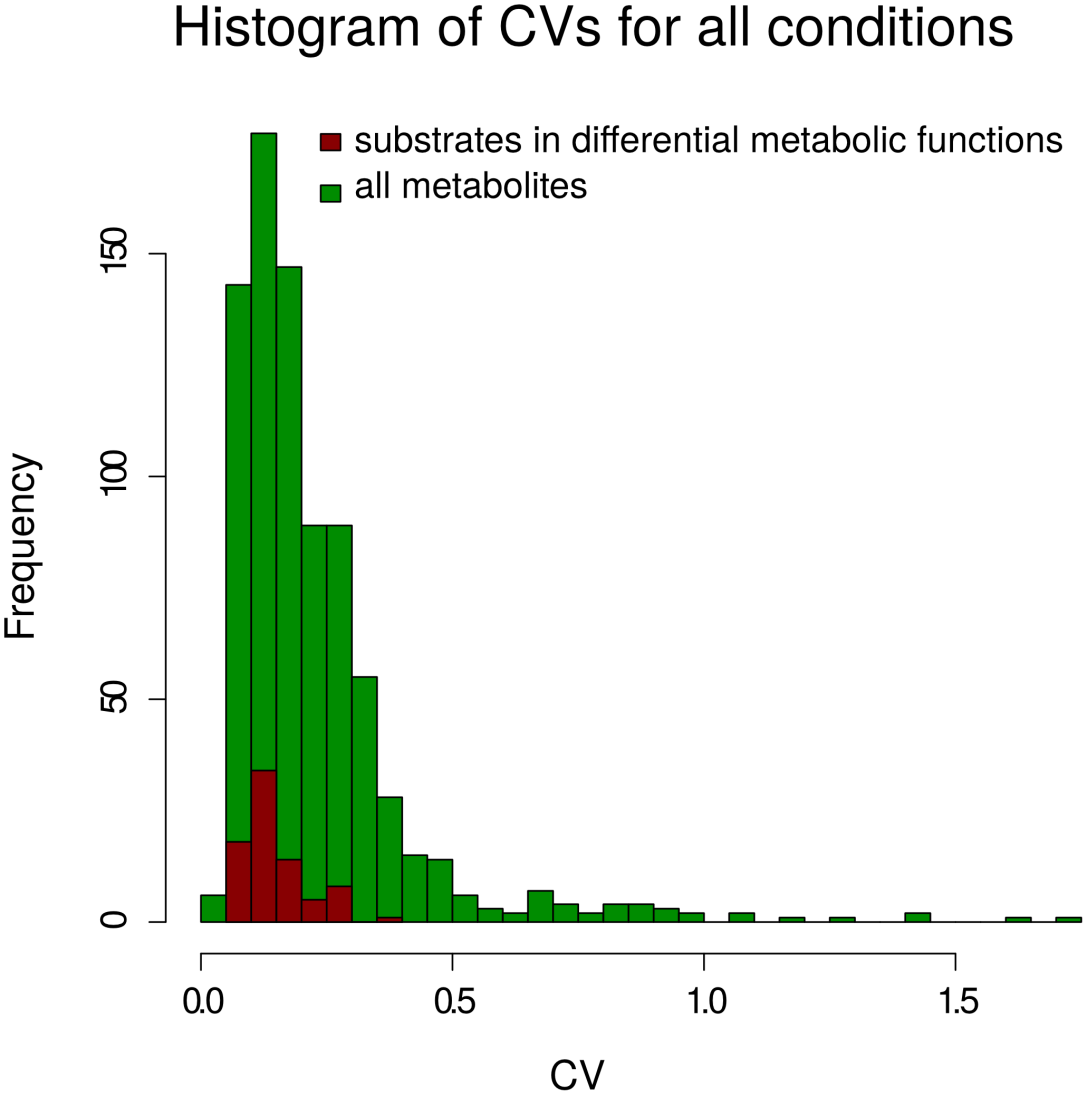
Histogram of temporal coefficient of variation for metabolites. Distribution of CVs over all measured metabolites (green) and all metabolites identified as substrates in a differentially behaving functions for the respective conditions (red). The plot summarizes the distributions over all eight considered conditions.

Figure 3 shows a histogram of the distribution of CVs for all measured metabolites and those that participate as substrates in the metabolic functions which were previously identified as sustainer or modulator over all eight investigated conditions. Histograms for each condition separately can be found in the Figure S2 in Supporting Information S2.

**Figure 4:**
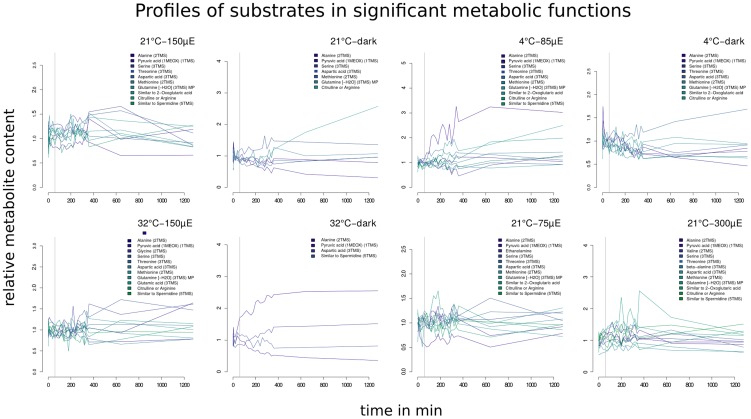
Time-series of the metabolites acting as substrates in differential metabolic functions. Profiles of the relative metabolite content of those measured metabolites that act as substrates in a pathway classified as differential with respect to the null model under the respective condition. The horizontal line indicates time point 60-organization and transition into a new metabolic state.

Inspecting the list of mapped metabolites, we identified 15 out of 65 to act as substrates in a differential pathway in at least one of the considered conditions, namely: alanine, pyruvate, serine, threonine, aspartate, methionine, glutamine, 2-oxoglutarate, citrulline or arginine, spermidine, glycine, glutamate, ethanolamine, valine, and 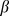-alanine. The temporal profiles of these metabolites for those conditions in which they act as substrates in modulator or sustainer are shown in Figure 4. All of these metabolites are either amino acids or essential intermediates in central carbon or nitrogen metabolism. The differential pathways they belong to fall into the larger groups of primary nitrate assimilation (glutamate, glutamine, and 2-oxoglutarate), photorespiration (glycine, serine, and ethanolamine), TCA cycle (pyruvate and 2-oxoglutarate), amino acid metabolism (alanine, arginine, threonine, aspartate, methionine, and valine) and polyamine biosynthesis (spermidine and 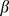-alanine). A discussion about the involvement of the respective differential pathways in stress responses to the eight environments investigated was already given in [23]. Apart from their involvement as substrates in modulators and sustainers, these metabolites have also been implicated in various other stress responses, *e.g.*, anoxia [34] or hypoxia [35], oxidative stress [36], drought stress [37], or general stress responses [38], [39].

Additionally, we tested if the described patterns of robustness in the metabolite profiles can also be found in the flux capacity profiles of the differential metabolic functions they belong to. Interestingly, only for 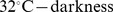 we also observe the flux capacity profiles of the differential pathways to exhibit significantly lower CVs than the non-differential pathways (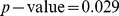). Another general observation that we made is, that for all metabolic functions for which substrate measurements were available, the CVs of these substrates were significantly lower than the CVs of the respective flux capacity profile. These two observations further underline the none-trivial interconnection between flux rates and the levels of metabolites.

### Substrates in differential metabolic functions are more connected

Next, we investigated whether the group of substrates in differential metabolic functions shows distinct characteristics with respect to the network topology. For the analysis we neglected evidently ubiquitous cofactors, such as: 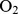, 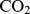, 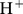, ADP, ATP, 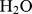, 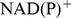, NAD(P)H, CoA, (pyro-)phosphate. This strategy has also been followed in other studies [40]. Furthermore, to arrive at a value for the connectivity of each metabolite, *i.e.*, the number of reactions in which a metabolite is involved (as defined in [41]), we kept the compartmentalized structure of the network and considered the instances of a metabolite appearing in more than one compartment as different reactants. Based on the given stoichiometry we determined the number of reactions in which each metabolite participates. Interestingly, we find the group of measured substrates of differential pathways to be on average significantly more connected than the group of all metabolites—6.24 vs. 2.99 reactions (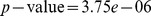, *Wilcoxon* rank-sum test). Clearly, one has to keep in mind that our analysis is based on a generic compartmentalized network reconstruction. The connectivity values might vary in different tissues, due to the presence or absence of certain pathways. Nevertheless, we belief that the well-curated model we use in our study serves a good starting point for the analysis.

### Differential metabolic functions have less substrates

To further investigate which attributes are typical for differentially behaving pathways, we next investigated the number of (initial) substrates of the pathways. Cofactors of the considered reactions were neglected from the analysis (see Supporting Information S3). Counting the number of substrates, we found their average number in the differential pathways to be significantly smaller in comparison to all considered pathways/all non-differential pathways (2.6 and 3.3/3.5 substrates, 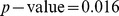/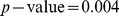, respectively). In contrast to this, when considering the number of initial substrates, we found that 29.7% of differential pathways have two initial substrates, while the remaining ones have only one substrate. In the whole group of metabolic pathways and the group of non-differential pathways this value is lower (25.5% and 26.2%, respectively) although not significant.

## Discussion

In this study, we extended our earlier analysis of *Arabidopsis*'s metabolic acclimation to varying light and/or temperature conditions which was based on transcriptomics data [23], [24]. Here we considered metabolomics data from the same experiment and investigated the temporal variation of the metabolite profiles. Our findings from the integrative analysis include the following: (*i*) for specific environmental conditions, differential metabolic functions have substrates, which on average show a lower CV than other metabolite groups tested, (*ii*) when considering the network topology, these substrates are on average more connected than the remaining metabolites and (*iii*) differential metabolic pathways have on average fewer substrates than the other metabolic functions investigated.

Closer inspection of the environmental conditions that exhibit low substrate variability leads to the following hypothesis: substrate robustness can be observed under stressful environmental conditions. Yet, we do not observe substrate robustness, or in genetic terms canalization, under conditions which are not perceived as stress by the plant (*e.g.*, 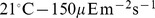) and moreover the canalization effect might get lost under those conditions which are too extreme or prolonged (*e.g.*, 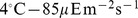). The latter scenario might cause a serious disturbance of the acclimation which could potentially lead to non-resilience, *i.e.*, non-recovery. Therefore, we believe that the observed substrate robustness is an inducible genetic mechanism, both depending on the metabolic network structure and the specific environmental condition. Determining the range of conditions that permit the observed robust behavior would be an interesting undertaking for future experimental testing.

Deriving flux values from transcriptomics data is a delicate issue. In recent years, a large set of methods have been proposed that use transcriptomics data to infer condition-specific networks, mainly applied on microorganisms (GIMME, [42], iMat [43], E-flux [16], PROM [44], MADE [45], TEAM [46]). While most approaches rely on a discretization of the expression data and employ user-defined thresholds, the here applied E-flux method does not rely on these requirements. It assumes a relationship between the amount of a certain transcript and the upper flux boundary of the respective reaction. While mechanisms, such as post-transcriptional modification and hierarchical regulation [47], [48] cannot be explicitly considered, they are implicitly accounted for by only restricting the upper flux boundary. In other words if a certain amount of transcript was measured the predicted flux can range between zero and the upper boundary; no enforcements on certain minimum flux values are made. Additionally, claims are even made with more reservation since the approach does not attempt to predict actual fluxes but flux capacities that are compliant with the data. Moreover, one needs to keep in mind that the employed metabolite data are not compartment-specific. In the analysis presented here, we assigned the same metabolite profile to each compartment-specific compound in the model. It would be interesting to investigate in future studies, when more compartment-or tissue specific metabolite data become available, if the observed patterns of substrate robustness are not only specific for certain environments, but also for particular compartments or tissues.

Finally, like any other modeling attempt, any results depend on the quality of the network as well as on the quality of the collected data. Here, we relied on the most recent and most comprehensive network reconstruction of *Arabidopsis* and a dataset that was collected with a single technology in a single laboratory to minimize technical artifacts.

### Comparison to related studies

The principle of keeping levels of metabolites involved in important pathways from exhibiting fluctuations was recently discussed in another context. Reznik at al. used the dual formulation of a classical FBA problem, which uses the maximization of biomass as a cellular objective, to compute sensitivities of the objective value to flux imbalances, *i.e.*, deviations from the steady-state assumption [49]. The so-called shadow price of a given metabolite captures the influence of the metabolite's accumulation or depletion on the maximum value of the objective. Thereby, a negative shadow price implies that the corresponding metabolite is growth limiting. By using data from *S. cerevisae* under different nutrient limiting conditions the authors were able to show that the determined shadow prices negatively correlate with the growth limitation of the respective measured intracellular metabolites. Moreover, based on these findings, the authors argued that growth-limiting metabolites cannot exhibit large fluctuations. Using data from *E. coli*'s metabolic response to carbon and nitrogen perturbations, they further demonstrated that metabolites associated with a negative shadow price indeed show lower temporal variation in comparison to metabolites with zero shadow prices in a perturbed system.

What both approaches, ours and the one briefly described above, have in common is the principle that metabolites important for a particular function exhibit less temporal variation than other metabolites. In the latter, an important metabolite is defined as a metabolite with a negative shadow price with respect to the assumed cellular objective of growth maximization. In contrast to this, our analysis is driven by integration of transcriptomics data and does not assume a particular overall cellular objective. In our approach, we consider a metabolite relevant if it acts as a substrate in a metabolic pathway which behaves differentially in comparison to a condition-specific null model for flux capacities. These relevant metabolites may thus play a role in the plant acclimation to environmental changes.

### The role of substrates of differential metabolic functions in network topology

Additionally to the observed substrate robustness in differential pathways under certain abiotic stress conditions, we also showed that these substrates are on average more connected *i.e.*, involved in more reactions. The role of these highly connected metabolites has previously been discussed in terms of evolution [40], [50]. In the latter, the authors identified among others, pyruvate, serine, aspartate, 2-oxoglutarate, and glutamate and put forward the hypothesis that the most highly connected metabolites should also be the phylogenetically oldest [40]. The connection between metabolites involved in core reaction of central carbon metabolism and their involvement in abiotic stress acclimation, together with the observation that they are on average more connected, extends this concept and puts the evolutionary structure of metabolic networks into a more dynamic context—one which also accounts for the changing environments affecting the organism.

### The role of substrates as systems input in terms of number and variability

Our third finding concerning the smaller number of substrates in differentially behaving metabolic functions has wide implications on the interplay between plasticity and robustness in metabolism. Most notably, our findings differ from claims made with respect to evolution of robustness and cellular stochasticity of gene expression. In a recent study, the author proposed that the degree to which varying cellular components combine to determine robust phenotypes may be predictive of the amount of their inherent variability. The basis for this claim is the observation that averaging over multiple independent inputs is a general way to reduce variability of molecular phenotypes [51]. This implies that the larger the number of variable inputs is, the smaller the variability of the phenotypic output will be. However, this observation does not apply to metabolic reactions which are governed by multiplicative (*e.g.*, mass action, as the simplest) rather than averaging laws. Here we observe that fewer input variables with smaller fluctuations, do not necessarily result in smaller fluctuations of the output (*i.e.*, the flux capacity in our case) but in robust differential behavior. Furthermore, our findings also showed that for all metabolic functions for which substrate measurements are available, the CVs of their substrates are significantly lower than the CVs of the respective flux capacity profile. This further highlights the particularities of regulation, variability, and robustness of metabolic pathways.

### Further biological implications

Robustness of certain pathway fluxes and specific metabolite concentrations have long been documented. The concept of network rigidity has initially been proposed in *S. cerevisae* [52]. Subsequently it has been demonstrated to be functional in plant systems too, especially in the context of central metabolism [53], [54]. Moreover, considered evidence has also accrued for certain metabolite levels to be exceptionally stable, for example the levels of alanine, pyruvate, 2-oxoglutarate, glutamine and spermidine [55], [56]. Furthermore, it has been shown that levels of metabolites such as serine coordinately control the level of expression of genes which encode multiple steps of the pathway in which they themselves take part [57].

In our view, the high stability of a pool of primary metabolites, invariant to environmental heterogeneity, fulfills two major functions. On the one hand, it efficiently sustains a set of “core” reaction rates which are deemed essential for the plant's objective function across a wide range of different stresses. On the other hand, the observed substrate stability enables a tight conditional control on a set of metabolic functions to act as modulators or sustainers in response to specific stresses only.

Finally, the fact that the robust metabolites may well be the most biologically relevant for metabolic regulation is an important point since it is at odds with the manner in which the majority of the metabolomics community assesses their data. This fact additionally highlights the potential difficulties and challenges in interpreting data from a single level of the cellular hierarchy and thus provides further grounds for integrated models such as the one we present here.

Taken together, our findings show that the integration of large-scale modeling with high-throughput data can be used to infer regulatory principles from the stoichiometry of the underlying reactions alone. Furthermore, we presented an approach that bridges the gap between flux-centric and metabolite centric view of large-scale data. Therefore, our study paves the way for investigating the existence of similar principles relating plasticity of metabolic profiles and robustness of metabolic behavior across other species for which both genome-scale networks and high-throughput (time-resolved) metabolomics data of high quality are becoming increasingly available.

## Materials and Methods

### Data

The investigated data set captures the time-resolved response of *Arabidopsis thaliana* to changing light and/or temperature conditions [26]. The previously published data comprise time-series measurements for eight environmental conditions covering combinations of four different light intensities (ranging from high-light (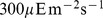) to darkness) and three different temperatures (4, 21, and 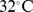). A schematic representation of the combinations of abiotic stresses is provided in the Figure S1 in Supporting Information S2. In brief, wild-type *Arabidopsis thaliana* Columbia-0 plants were grown in soil under short-day-conditions for 4 weeks and then transferred to long-day-conditions for another 2 weeks. Subsequently, they were exposed to the following conditions: 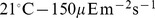; 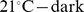; 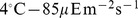; 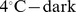; 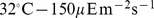; 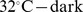; 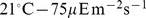, and 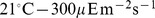. Metabolite and transcript profiles were collected from samples harvested at 22 time points ranging from 0 to 24 hours after the stress application. Further details of the experimental procedures and data processing can be found in the original publication [26]. Transcriptomics data are deposited in the array express repository (http://www.ebi.ac.uk/arrayexpress) under Arabidopsis light and temperature response ArrayExpress accession: E-MTAB-375 and they can be downloaded using the following link: http://www.mpimp-golm.mpg.de/Supplementary-Materials-for-Publications/Caldana-et-al_Filtered-Affymetrix-Gene-Expression-Data.zip. Metabolomics data are provided on the following website http://www.mpimp-golm.mpg.de/Supplementary-Materials-for-Publications/Caldana-et-al_Normalized-metabolic-data.zip.

### Mapping of high-throughput data onto the model

From the total of 82 measured metabolites, 65 can be mapped onto the model. It must be noted that the metabolomics data are not compartment-specific and 61 out of 65 mapped metabolites appear in more than one compartment in the model. Due to a lack of this information, for those non-unique metabolites we assigned the same profile to each compartment-specific compound in the model. The mapping dictionary is given in the Supporting Information S4.

The mapping of the transcriptomics data from our previous study has a network coverage of 46%, *i.e.*, 627 out of 1363 reactions can be constrained by transcriptomics data [23], [24].

### Flux Balance Analysis

FBA is a constraint-based approach for predicting steady-state fluxes in a metabolic network independent of enzyme kinetics and metabolite concentrations [10], [11]. The method solely relies on the physico-chemical constraints of the network (*e.g.*, the reaction stoichiometry, reversibility, and maximum uptake rates) and a putative biological objective of the organism under consideration (*e.g.*, biomass production for microorganisms under ambient conditions). A central element of the approach is the assumption of a steady-state which implies that each internal metabolite 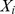 in the network is produced and consumed at the same net rate if considering the system at a small time interval, or in a mathematical representation: 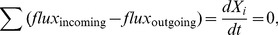
(1)which results in a decoupling of the flux predictions from the metabolite concentrations. Adding the above mentioned additional constraints and assumptions leads to the following linear program:
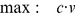
(2)

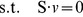
(3)

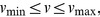
(4)where 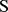 is the stoichiometric matrix of the system under consideration in which the rows denote the metabolites 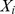 and the columns represent the reactions of the model. The reaction fluxes are captured in the flux vector 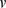. The respective lower and upper boundaries of the reaction are given by 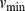 and 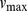. The vector 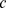 encodes the ratios at which certain precursors (*e.g.*, amino acids, fatty acids, nucleotides, sugars) contribute to the objective function. For a detailed review of FBA and other related constraint-based optimization approaches see [11], [13].

### Metabolic functions

The analysis presented here extends results recently presented in [23]. In brief: In our previous study we had simulated flux capacities through a set of a 167 metabolic functions. The simulation of metabolic functions has initially been proposed to demonstrate the quality of a metabolic reconstruction [58] and it has also been used in the original model reconstruction [25] to ensure model functionality. The set of selected pathways cover primary as well as secondary metabolism and is obtained from AraCyc/MetaCyc [59]–[61]. These databases incorporate community-wide efforts to integrate current biological knowledge and understanding of metabolic pathways. In our previous study we extended the proposed concept to simulate time-and condition-specific flux capacities by integrating transcriptomics data based on a modification of the E-flux method [16], which assumes a relationship between the amount of a certain transcript and the upper flux boundary of the respective reaction. Since the correspondence between transcript and protein abundance is crucial when using transcriptomics data to constrain flux boundaries, the approach only makes weak assumptions. Proteins are allowed to be present and active if the respective gene product is detected. In contrast, no enforcements on protein activities are made if the gene product was detected with certain abundance. Additionally, our claims are stated with even greater reservation since the approach does not attempt to predict actual fluxes but flux capacities that are compliant with the data. For details of the simulation and the list of metabolic functions refer to [23], [25].

### Statistical methods

To determine the temporal variation of the metabolite profiles we used the coefficient of variation (CV) which is defined as the ratio of the standard deviation 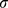 and the mean 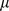 of the observable: 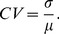
(5)


We applied this statistic to the mapped metabolite profiles for each condition separately. While doing so, we neglected the data for the first hour (first six time points) after the stress application during which the system experiences the strongest effect, *i.e.*, differential regulation of pathways involved in the response. To ensure that the categorization of differential metabolic functions is robust to the removal of this time-interval we repeated the analysis of our previous study. Reassuringly, we find our results, *i.e.*, the classification as a sustainer or modulator of a given metabolic state, to be robust to the removal of up to six time points from the beginning of the time series.

## Supporting Information

Supporting Information S1 **List of tested metabolites.** Given is the number of metabolites in the respective group for each condition on which the *Wilcoxon*-test is based.
(XLS)

Supporting Information S2 **Additional figures.** Figure S1: Schematic representation of the eight environmental conditions. Plants were grown under ambient conditions and then transferred into one of the eight indicted conditions. Figure S2: Condition-specific distribution of the coefficients of variation (CVs) for all mapped metabolite profiles. Given are the distributions of CVs for all measured metabolites (green) and those that participate as substrates in the metabolic functions that were previously identified as sustainer or modulator (red) for all eight investigated conditions separately.
(DOC)

Supporting Information S3 **Metabolites regarded as cofactors or constant and neglected from the analysis.** Given is the Metabolite ID, the AraCyc/PMN ID, the KEGG ID, the metabolite name, and the formula.
(XLS)

Supporting Information S4 **Metabolite dictionary.** Given is the mapping of the metabolites between that data and the model. Some metabolites have two possible metabolites IDs in the model. Note that a metabolite ID is not unique and the same metabolite can participate in reactions in different compartments.
(XLS)
