# Supplementary material for: Variability of Metabolite Levels Is Linked to Differential Metabolic Pathways in Arabidopsis's Responses to Abiotic Stresses
Source: PLoS Comput Biol. 2014 Jun 19;10(6):e1003656. doi: 10.1371/journal.pcbi.1003656 (PMC4063599; doi:10.1371/journal.pcbi.1003656)
Supplement: Supporting Information S2 — Additional figures. Figure S1: Schematic representation of the eight environmental conditions. Plants were grown under ambient conditions and then transferred into one of the eight indicted conditions. Figure S2: Condition-specific distribution of the coefficients of variation (CVs) for all mapped metabolite profiles. Given are the distributions of CVs for all measured metabolites (green) and those that participate as substrates in the metabolic functions that were previously identified as sustainer or modulator (red) for all eight investigated conditions separately. (DOC) [file pcbi.1003656.s002.doc]

**Supporting Information S2**

**
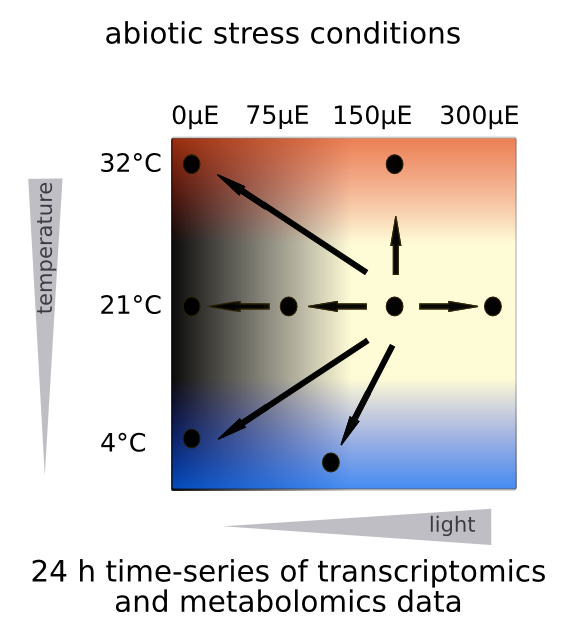
**

**Supporting Figure S1: Schematic representation of the eight environmental conditions**. Plants were grown under ambient conditions and then transferred into one of the eight indicted conditions.

| 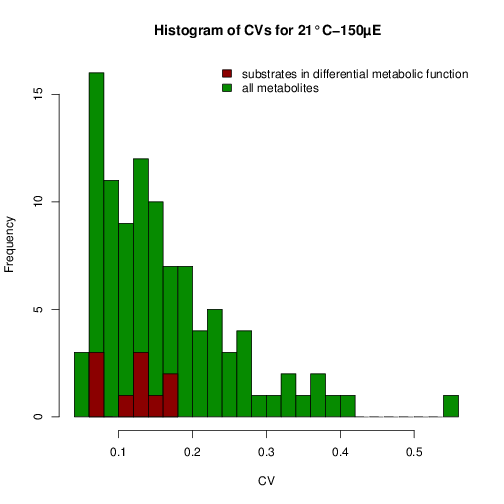 | 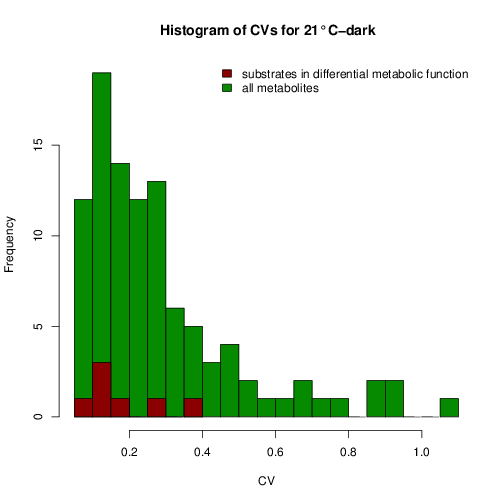 |
| --- | --- |
| 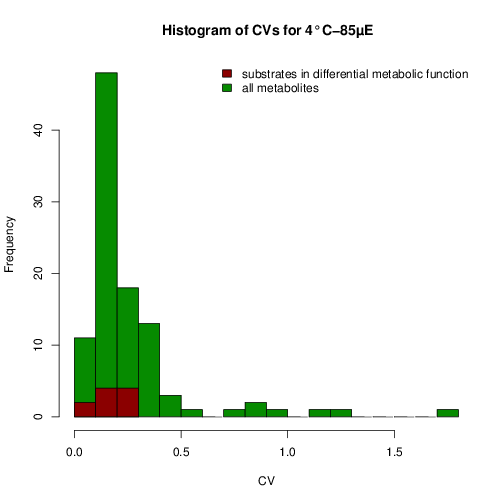 | 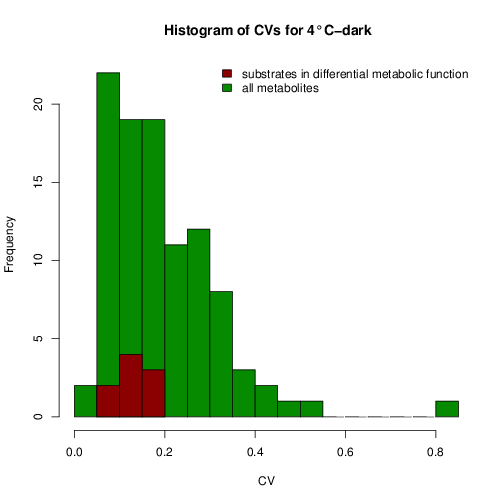 |
| 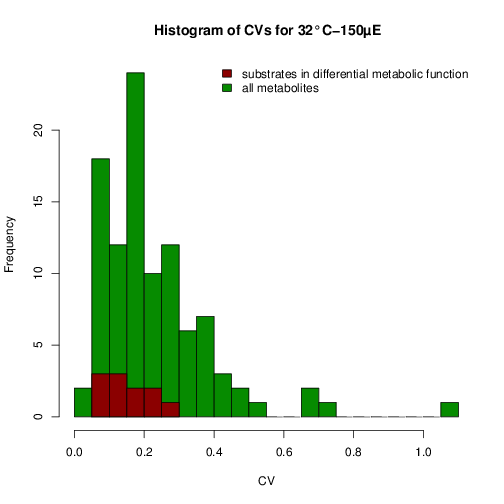 | 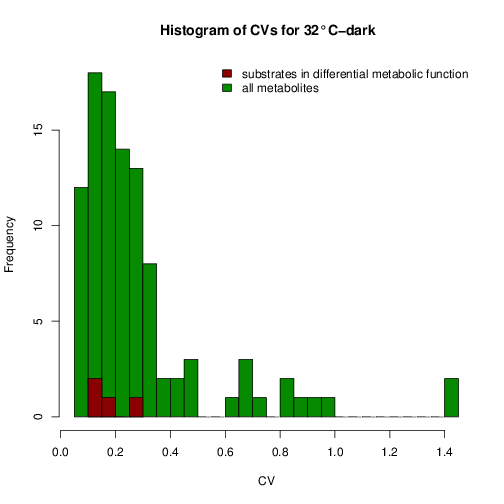 |
| 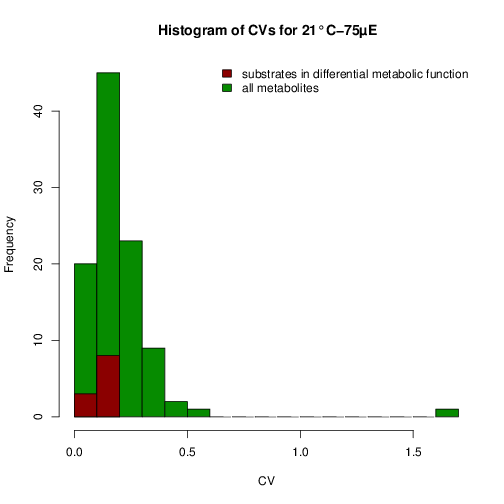 | 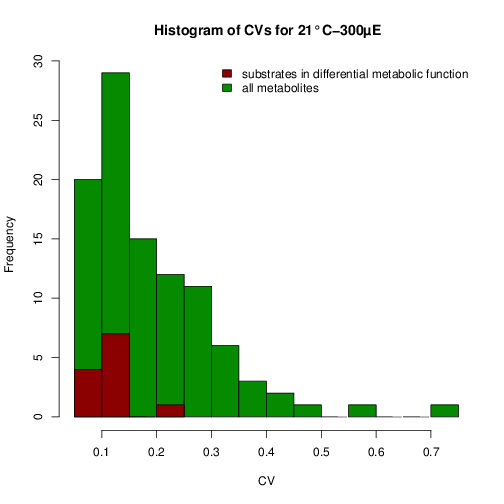 |

**Supporting Figure S2: Condition-specific distribution of the coefficients of variation (CVs) for all mapped metabolite profiles.** Given are the distributions of CVs for all measured metabolites (green) and those that participate as substrates in the metabolic functions that were previously identified as sustainer or modulator (red) for all eight investigated conditions separately.
